# Editorial: Advancing benign surgery: techniques, outcomes, and educational innovations

**DOI:** 10.3389/fsurg.2026.1851397

**Published:** 2026-05-12

**Authors:** Marina Yiasemidou, Alec Engledow, Dimitrios Damaskos

**Affiliations:** 1 Colorectal Surgery, The Royal London Hospital, Barts Health NHS Trust, London, United Kingdom; 2 NHS Lothian, University of Edinburgh, Edinburgh, United Kingdom

**Keywords:** benign, education, hernia, mental rehearsal, robotic, surgery, training

Benign surgery continued to represent a fundamental component of contemporary surgical care, encompassing procedures performed to treat non-malignant disease with the aim of improving symptoms, restoring function (1), and enhancing quality of life. Advances in surgical technology—particularly the expansion of minimally invasive and robotic-assisted techniques (2)—have substantially influenced the management of benign conditions across surgical subspecialties. Moreover, the increasing complexity of surgical innovation should also be mirrored with parallel developments in surgical training (3, 4) to enable current and future adaptation.

This Research Topic was developed to showcase contemporary progress in benign surgical practice by bringing together work on novel operative techniques, clinical outcomes, and educational innovation. It sought to bridge advances in clinical care with developments in surgical training, offering an opportunity to examine how these evolving approaches influenced patient selection, perioperative decision-making, technical performance, and workforce development. Collectively, the contributions aimed to inform best practice, improve patient outcomes, and support the ongoing refinement of surgical education in the context of benign disease.

A central focus of the topic was patient outcomes, encompassing strategies to prevent complications and to ensure their optimal management should they occur postoperatively. Wang et al. demonstrated that sarcopenia adversely affects recovery of gastrointestinal function after antireflux surgery, advocating routine screening and targeted prehabilitation. Zhao et al. recommended face mask ventilation to enhance preoxygenation and minimise gastric insufflation during laparoscopic cholecystectomy. Rebelo et al. suggested more restrictive use of biliary stents in choledocholithiasis due to associations with higher complication rates, while Khanal et al. emphasised individualised surgical strategies for complex ventral hernias to improve outcomes. Moreover, Addasi et al. have identified predictive factors for achieving critical view of safety (CVS) in theatre. The authors found that both preoperative and intraoperative factors can influence a surgeon's ability to achieve CVS. Lower ASA grade, emergency cholecystectomy, acute cholecystitis, and procedures performed by non-HPB surgeons were associated with a higher likelihood of attaining CVS. They further suggested that standardised protocols and structured training may improve both the achievement and documentation of CVS across diverse practice settings. Wyland et al. explored the use of mesh in laparoscopic hiatal hernia repair. They found that it was associated with increased operative time, length of stay, and hospital readmission. There were no differences in mortality or overall morbidity. Case reports by Bourouail et al. and Dionysios et al. examined the management of hypopharyngeal fistula post-open diverticulectomy for giant Zenker diverticulum and giant incisional hernia repair, respectively.

New technologies or new applications of existing ones were also explored in this topic. Chen et al. assessed the feasibility and outcomes of robotic assisted partial splenectomy, reporting good results. Jeong et al. examined the learning curve for single-port laparoscopic appendicectomy. The authors reported that competence was achieved after the 13th case, while mastery was attained after the 36th. They suggested that, provided surgeons have sufficient prior laparoscopic experience, Single Port Laparoscopic Appendicectomy can be introduced safely in low-volume settings, particularly when early cases are limited to young, healthy, non-obese patients. Diaz Casanova et al. investigated robotic surgery for multiple concurrent hernias, while Jayasinghe et al. described methods to intraoperatively identify ureters to prevent injury. Yang et al. assessed the outcomes of V-NOTES hysterectomy in obese patients and found it to be a safe approach, with outcomes broadly comparable to those in non-obese patients, albeit with longer operative and recovery times. Liao et al. described Intelligent pressure-controlled percutaneous unroofing of renal cysts and found it to offer shorter operative time, reduced postoperative hospital stay, and lower postoperative pain compared to laparoscopic, while maintaining similar safety profile and efficacy. They concluded that Intelligent pressure-controlled percutaneous unroofing may represent a superior minimally invasive option for the treatment of renal cysts. Youssef et al. debated the role of immunotherapy in the treatment of benign tumours as a targeted, non-surgical treatment strategy for benign tumors. Though predominantly applied to malignancies, immunotherapy may have untapped potential for treating benign tumors by exploiting tumor-specific antigens arising from defined genetic alterations. Recognising that benign tumors often cause significant morbidity and may remain unaddressed by current surgical and medical approaches, the authors discuss how modalities such as immune checkpoint inhibitors, adoptive transgenic T-cell therapies, and bespoke vaccines might be repurposed or developed to target these antigens effectively. Benign tumor profile may favour sustained immune engagement; the authors outline preclinical evidence, potential targets, and combinatorial strategies designed to maximise efficacy while managing safety considerations. The review ultimately positions immunotherapeutics as a promising paradigm to expand treatment options and improve clinical outcomes for patients with benign neoplasms.

Emphasis was also placed on the use of advanced recovery techniques to enhance patient outcomes. Mu et al. conducted a multicentre cross-sectional study assessing adherence to the enhanced recovery after surgery (ERAS) protocol and its influencing factors among patients in south-western China. The authors found that adherence remains suboptimal, with rates affected by hospital tier, patients' educational background, perceptions and attitudes towards ERAS, and the extent of social and environmental support. Wang et al. reviewed the implementation and outcomes of ERAS in the perioperative care of gynaecological patients, highlighting its benefits across both benign and oncological procedures. Their synthesis demonstrated that ERAS is associated with reduced length of hospital stay, lower complication rates, accelerated functional recovery, decreased pain scores and opioid use, and increased same-day discharge rates. Persistent barriers to uptake, including variability in implementation, clinician and patient perceptions, and systemic constraints, were also identified. The review emphasises that, while ERAS clearly improves perioperative outcomes, the adoption of standardised protocols, targeted education, and multidisciplinary engagement is essential to fully realise its potential.

In addition to these clinical and technical advances, the topic also addressed important aspects of surgical training. Berg et al. assessed whether internationally validated methods exist for training and evaluating surgical skills. Existing evidence was found to be fragmented, procedure- and specialty-specific, with limited scientific validation and poor global generalisability. No internationally validated, universally applicable framework for manual surgical skills training and assessment was identified. They advocate for a clear need for simple, reliable, and internationally comparable methods to assess surgical technical competence across diverse healthcare settings. Walshaw et al. provided a comprehensive narrative review on mental rehearsal, detailing its definition, applications, current evidence, and practical guidance for integration into surgical practice.

In summary, this Research Topic highlights the breadth and dynamism of contemporary benign surgical practice, demonstrating how innovation in operative technique, perioperative care, and complication prevention continues to shape the management of non-malignant disease. Across a diverse range of contributions, the collection underscores the importance of careful patient selection, evidence-based technical refinement, and the thoughtful adoption of emerging technologies, including minimally invasive, robotic, and novel non-surgical approaches. Collectively, these studies reinforce the principle that progress in benign surgery must remain firmly anchored in the pursuit of improved patient outcomes, safety, and quality of life.

Equally, this body of work emphasises that advances in benign surgical care cannot be considered in isolation from developments in surgical education and training. As procedures become increasingly sophisticated and expectations around safety, standardisation, and technical performance continue to rise, robust and adaptable training frameworks will be essential to support both current and future surgical practice. Taken together, the contributions in this Research Topic not only reflect the evolving landscape of benign surgery, but also provide a valuable foundation for ongoing research, innovation, and educational development in the field.
